# Current definition, diagnosis, and treatment of canine and feline idiopathic vestibular syndrome

**DOI:** 10.3389/fvets.2023.1263976

**Published:** 2023-09-22

**Authors:** Anna Morgana Mertens, Henning Christian Schenk, Holger Andreas Volk

**Affiliations:** ^1^Department of Small Animal Medicine and Surgery, University of Veterinary Medicine Hannover, Hanover, Germany; ^2^Department of Neurology/Neurosurgery, Tierklinik Lüneburg, Lüneburg, Germany

**Keywords:** veterinary neurology, vertigo, idiopathic vestibular disease, definition, diagnosis, treatment

## Abstract

Idiopathic vestibular syndrome (IVS) is one of the most common neurological disorders in veterinary medicine. However, its diagnosis and treatment varies between publications. The aim of the current study was to gather experts’ opinion about IVS definition, diagnosis, and treatment. An online-survey was used to assess neurology specialists’ opinion about the definition, diagnosis and treatment of IVS. The study demonstrated that the definition, diagnosis, and treatment of IVS are largely consistent worldwide, with the EU prioritising less frequently advanced imaging and more often otoscopy to rule out other diseases. IVS was defined by most specialists as an acute to peracute, improving, non-painful peripheral vestibular disorder that often affects cats of any age and geriatric dogs. Regarding diagnosis, a detailed neurological examination and comprehensive blood tests, including thyroid values, blood pressure, and otoscopic examination, was seen as crucial. A thorough workup may also involve MRI and CSF analysis to rule out other causes of vestibular dysfunction. Treatment of IVS typically involved intravenous fluid therapy and the use of an antiemetic, with maropitant once daily being the preferred choice among specialists. Antinausea treatment was considered, however, only by a handful specialists. This survey-based study provides valuable insights from neurology experts and highlights areas that require further research to bridge the gap between theory and practice.

## 1. Introduction

Idiopathic vestibular syndrome (IVS) is a common neurological disorder observed in dogs over the age of nine, elderly cats, and cats with any age with certain regions in North America experiencing seasonal patterns (1–3). Clinical manifestations of IVS typically manifest acutely, with affected animals displaying a head tilt towards the side of the lesion, pathological nystagmus, positional strabismus, and vestibular ataxia. Nausea and emesis may also occur (1, 3–7). The majority of cases show improvement within a couple of days, and complete resolution of clinical signs is typically observed within 2 to 4 weeks. However, mild residual clinical signs, such as a slight head tilt or mild ataxia can persist in some cases for life (1, 3, 4, 6). The rapid improvement of clinical signs is a distinguishing factor that sets IVS apart from other causes of vestibular disease (3, 8). As IVS is a diagnosis of exclusion, albeit no common consensus exist, it has been proposed by authors to require an extensive diagnostic workup to rule out other potential causes, including cerebral ischaemia, otitis media/interna, meningoencephalitis, trauma, thiamine deficiency, tumors, and other conditions (1, 3, 4).

The pathomechanism of IVS in veterinary medicine remains incompletely understood. In human medicine, there are vestibular diseases that present with similar clinical signs, which have better understood aetiologies. For instance, Menière’s disease (MD) is characterised by distension of the membranous labyrinth of the inner ear, known as endolymphatic hydrops. Rupture of the endolymphatic hydrops disrupts the homeostasis of inner ear fluids, leading to episodes of vertigo, tinnitus, hearing loss, and a feeling of “stuffed ears” (8–12). Another related disorder is benign positional paroxysmal vertigo (BPPV), which involves the presence of free-floating otoliths in the semicircular canals of the inner ear. Free-floating otoliths stimulate sensory hair cells, generating false motion signals that cause vertigo (13–15). Acute vestibular neuritis (AVN) is another frequently encountered vestibular disorder in human medicine, often idiopathic in nature, however, has been associated with persistent herpes virus infection, autoimmune causes, or microvascular ischaemia. It is characterised by the sudden onset of dizziness lasting longer than 24 h, accompanied by nystagmus and nausea (13–17). Symptoms of AVN typically improve within a few days and completely resolve within weeks (16, 17).

Diagnosis and therapy for vestibular disorders in human medicine are tailored to its specific aetiology. A variety of treatment plans are available, ranging from symptomatic approaches to targeted therapies aimed at managing dizziness and promoting central compensation (18–20). One frequently prescribed medication is betahistine, a histamine derivative that improves blood circulation in the vestibular organ and enhances the functionality of the vestibular nuclei (21, 22). In addition to medication, special movement exercises are employed in human medicine. Different maneuvers are performed to address BPPV based on the affected semicircular canal. Specific rotational movements of the head facilitate the displacement of the otoliths from the affected canal into the utricle, relieving symptoms. Coordination exercises are also used to accelerate compensatory mechanisms in the brain and improve clinical signs of vertigo (23). In contrast, in veterinary medicine there are currently no clear guidelines for diagnosis and treatment, some authors have recommended a symptomatic therapy approach for IVS, which traditionally includes the use of antinausea medications (e.g., ondansetron), antiemetics (e.g., maropitant, metoclopramide), and intravenous fluid therapy (3, 4, 7, 24).

The aim of the current study was therefore to capture veterinary neurology experts’ opinion about the definition of IVS, their preferred diagnostics and therapeutics in dogs and cats with IVS. The second aim was to see if there are regional differences in expert opinions. The results of the study could inform clinical practice, but more importantly future research into IVS, which is an under-researched area.

## 2. Materials and methods

### 2.1. Survey design

A survey was conducted using the LimeSurvey® online platform (Hamburg, Germany) to gather data from specialists in veterinary neurology, specifically targeting ACVIM (Neurology) and ECVN board-certified veterinarians. To recruit participants, the survey link was disseminated on the Veterinary Information Network®, Inc. (Davis, California, USA) online platform, list servers and through social media channels. The survey aimed to gather information on the specialists’ expert opinion regarding IVS, including their self-written definitions of the condition and any potential new specifications derived from their clinical experience.

The survey employed a combination of free-text and multiple-choice questions to gather data on the preferred diagnostic procedures for dogs and cats. The multiple-choice questions were supplemented with a free-text response option to capture any diagnostic procedures not listed in the predefined options. The question on preferred diagnostic methods was divided into two parts. In the first part, participants could select multiple answers to indicate the complete diagnostic approach for IVS. The second part limited participants to selecting only five answers, enabling identification of the most important diagnostic procedures for each specialist. The complete questionnaire can be found in Supplementary data A. Treatment preferences were also assessed using a multiple-choice question, accompanied by an additional free-text response option, for both dogs and cats. Participants were not restricted in the number of responses they could provide, allowing for a comprehensive overview of the therapies utilised in practice.

To evaluate the definitions provided by participants, a modified qualitative evaluation method based on Mayring’s approach was employed. Keywords from the definitions were extracted and tallied, and a Word Cloud was generated to visualise the frequency of these keywords (25). The collected data pertaining to diagnosis and therapy were recorded and analysed using Microsoft 365 Excel (Microsoft Corporation, Redmond, Washington, USA). Participants were categorised into geographic groups, including North America, Europe, and the UK, to examine any regional preferences.

## 3. Results

### 3.1. Participant’s demographic

One-hundred-seventy-seven neurologists participated in the online survey (North America (NA) 63/177, 36%; European Union (EU) 42/177, 24%; United Kingdom (UK) 22/177, 12%) of which 50 neurologists (28%) did not specify their country. One-hundred-two formulated a definition of IVS (NA 53/102, 52%; EU 34/102, 33%; UK 15/102, 15%) and 112 specialists (NA 59/112, 53%; EU 37/112, 33%; UK 16/112, 14%) filled in the second part of the survey, containing the different diagnostic options for IVS. The treatment modality question of the survey was completed by one-hundred-seven participants (NA 55/107, 51%; EU 36/107, 34%; UK 16/107, 15%; Supplementary Table 1).

### 3.2. Definition of IVS

The onset of clinical signs was most frequently described as “acute” to “peracute” (*n* = 53/103, 51%). Almost half of the participants did not characterise the time of onset (*n* = 50/103, 49%). Different words for the course of the clinical signs were used by more than half of the participants (*n* = 29/103, 28%; “Improve/improvement” *n* = 15/103, 15%; “resolve/resolution” *n* = 8/103, 8%;"non-progressive” *n* = 6/103, 6%). The most commonly mentioned word for the neuroanatomic localisation was “peripheral” (*n* = 71/103, 69%). Other words used were “unilateral” (*n* = 8/103, 8%), “no central involvement” (*n* = 7/103, 7%) and “bilateral” (*n* = 2/103, 2%). Some participants (*n* = 15/103, 15%) did not specify the neuroanatomical localisation further. Affected patients were identified as “older” (*n* = 20/103, 19%). Most of the participants did not use a word to describe the signalment (*n* = 70/103, 68%). In some cases, IVS was referred to as a “disease” (*n* = 21/103, 20%), a “dysfunction” (*n* = 16/103, 16%) or a “disorder” (*n* = 4/103, 4%). Words used to paraphrase “idiopathic” were mainly “without cause” (*n* = 37/103, 36%). Fifty-seven specialists (*n* = 57/103, 55%) did not mention the idiopathic character of the disease.

**Figure 1:**
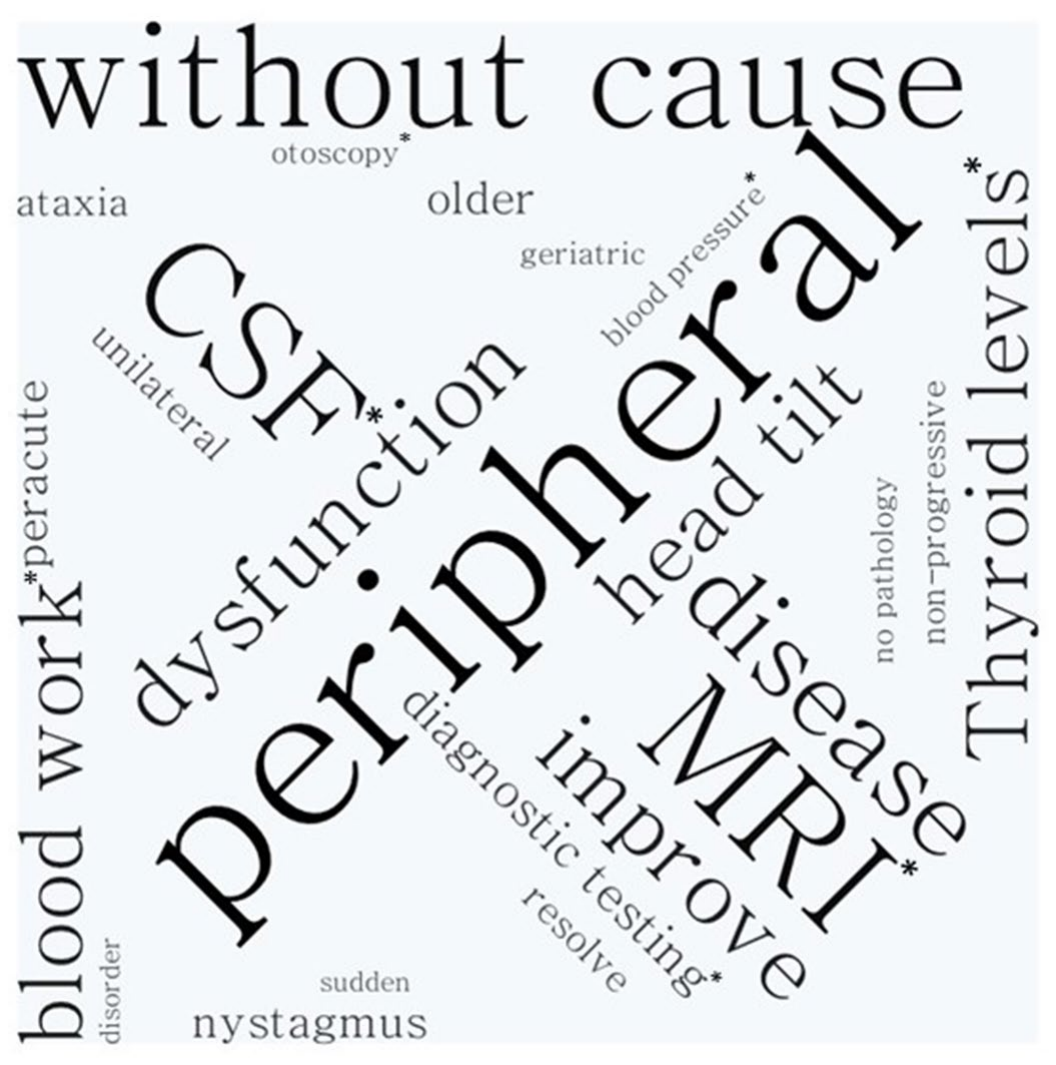
Word cloud analysis. As larger the word appears on the figure the more often it was used by the neurology specialist to describe idiopathic vestibular syndrome.

Participants mentioned in some of the definitions diagnostic tools that should be unremarkable for an IVS diagnosis. These included magnetic resonance imaging (MRI; *n* = 37/103, 36%), cerebrospinal fluid examination (CSF; *n* = 32/103, 31%), otoscopy (*n* = 5/103, 5%), blood pressure (*n* = 5/103, 5%), thyroxine (T4) and thyroid-stimulating hormone (TSH) serum concentrations (*n* = 13/103, 13%), and haematology and serum biochemistry (SB; *n* = 14/103, 14%). Some neurologists also listed specific clinical signs of IVS in their self-formulated definitions. “Head tilt” (*n* = 13/103, 13%), “nystagmus” (*n* = 11/103, 11%), “ataxia” (*n* = 8/103, 8%), “strabismus” (*n* = 1/103, 1%), and “balance disorder/deficit” (*n* = 3/103, 3%) were used in this context (Figure 1).

### 3.3. Diagnostic methods to identify IVS in dogs

**Figure 2:**
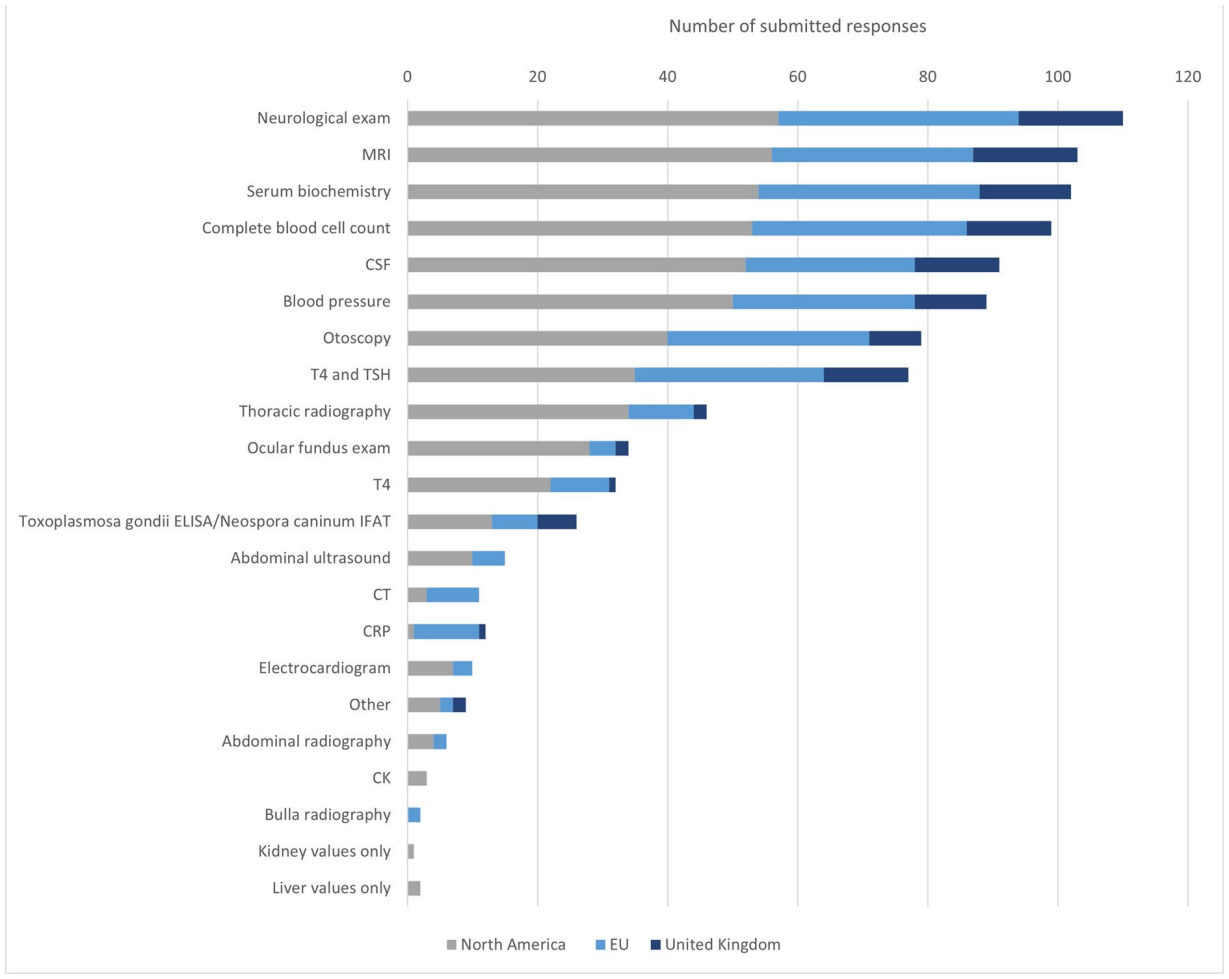
Diagnostic methods to identify IVS in dogs. MRI = magnetic resonance imaging, CSF cerebrospinal liquid examination, T4 = thyroxine, TSH = thyroid-stimulating hormone, ELISA = enzyme-linked immunosorbent assay, IFAT = Immuno-Fluorescence-Antibody-Test, CT = computer tomography, CRP = C-reactive protein, CK = creatine kinase.

The first diagnosis question aimed to define the most preferred diagnostics for the diagnosis of IVS. The participants were therefore not restricted in the number of choices they could choose from. One-hundred-twelve participants submitted their answers and named the following diagnostics essential for the diagnosis of IVS in the dog: neurologic examination (NE), MRI, SB, complete blood cell count (CBC), CSF examination, and blood pressure (BP) as essential for the diagnosis of canine IVS (Figure 2). Participants from NA listed especially MRI and CSF frequently. Specialists from the EU listed most commonly SB, CBC and MRI. Interestingly, for the EU participants otoscopy was selected as frequently as MRI, which was selected by a smaller percentage of the participants from the UK and NA. The five most frequently selected examinations of the UK participants are consistent with the overall group. In the UK, determination of T4 were selected notably more frequently in percentage and rank the same as CBC and CSF. BP and otoscopy, on the other hand, have lower mentions in the UK compared to the overall group, NA and EU. In the free text field, the following tests were also mentioned: urine test, urine protein creatine ratio, palpation for local bulla pain, free T4, BAER (brainstem auditory evoked response) and canine distemper titer (Figure 2).

### 3.4. Diagnostic methods to identify IVS in cats

**Figure 3:**
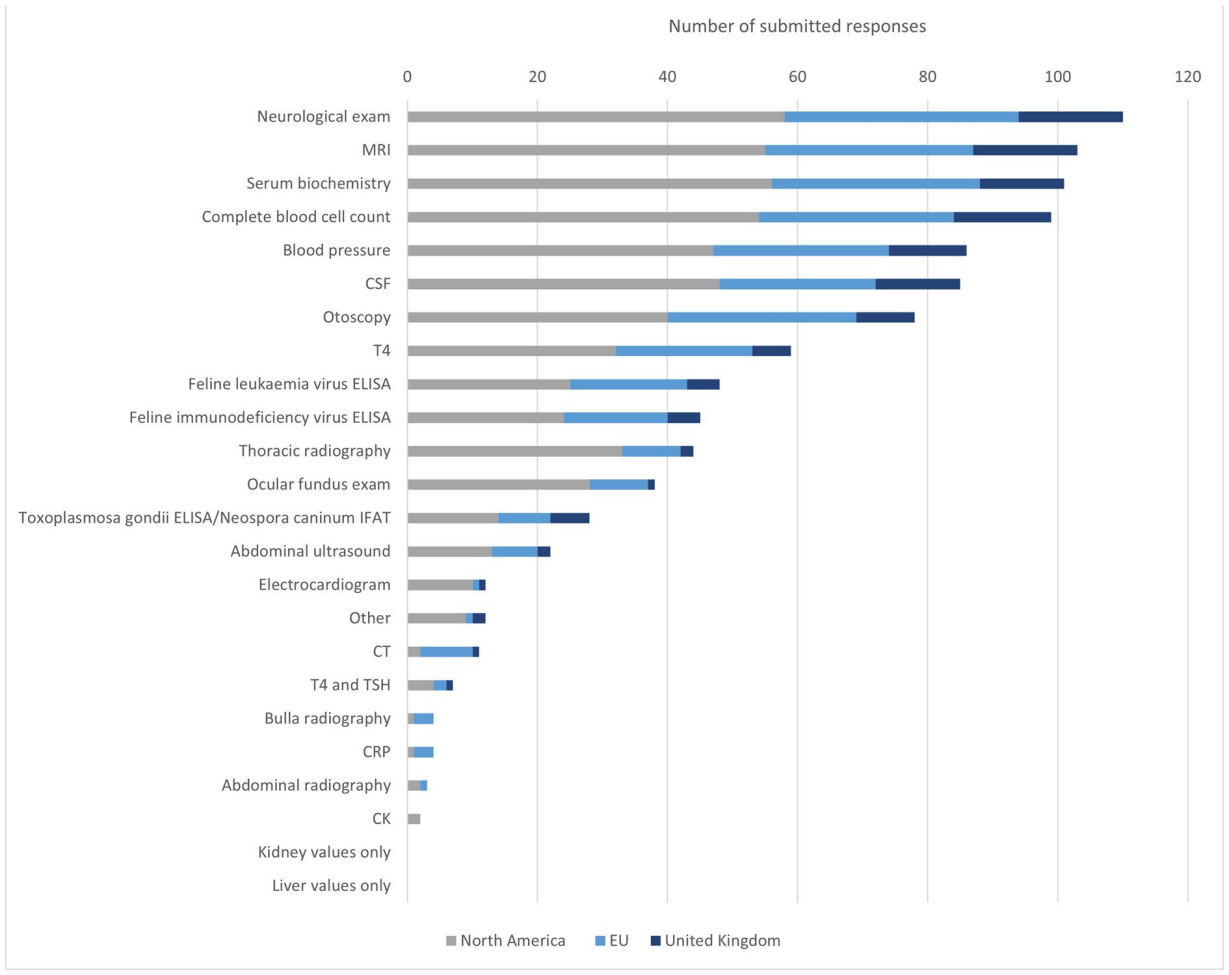
Diagnostic methods to identify IVS in cats. MRI = magnetic resonance imaging, CSF = cerebrospinal liquid examination, T4 = thyroxine, FeLV = feline leukaemia virus, FIV = feline immunodeficiency virus, ELISA = enzyme-linked immunosorbent assay, IFAT = Immuno-Fluorescence-Antibody-Test, CT = computer tomography, TSH = thyroid-stimulating hormone, CRP = −reactive protein, CK = creatine kinase.

The most frequently mentioned diagnostics for feline IVS were NE, MRI, SB, CBC, BP, and CSF. Compared to the overall group, SB was selected slightly more frequently by NA participants than MRI. NA specialist put a greater focus on the blood examination and SB than EU participants who selected both examinations 9% less (CBC NA *n* = 54, 94%; EU *n* = 30, 81%/SB NA *n* = 56, 95%; EU *n* = 32, 86%). On the other hand, otoscopy was selected 10% more often by EU than by NA respondents (NA *n* = 40, 68%; EU *n* = 29, 78%). EU participants selected otoscopy more often than BP and CSF relative to the overall group, NA, and UK. CSF was selected 11% less (all *n* = 85, 76%; EU *n* = 24, 65%). In the UK CSF was selected more frequently than BP. CBC was in addition selected considerably more often than SB with comparison to the overall group. MRI was picked by all participants, thereby being the most frequently selected diagnostic method in the UK, alongside NE. Cryptococcus titers, feline infectious peritonitis (FIP) diagnostics, urinalysis, palpation for bulla pain, and determination of thiamine levels were named in addition in the free text field (Figure 3).

### 3.5. The core diagnostic methods for IVS in dogs

When participants were forced to choose only five diagnostic tests the most chosen test was the NE, followed by an MRI, SB, otoscopy, and the determination of the thyroid levels T4 and TSH. NA neurologists placed greater emphasis, on the MRI, SB, CSF, CBC, BP, and measurement of thyroid levels T4 and TSH. EU participants in this survey prioritized beside NE otoscopy and T4 and TSH more often than an MRI. Neurologists from UK named MRI and analysis of T4 and TSH more frequently compared to the overall group. However, otoscopy and SB were selected not as often. The data can be found in the Supplementary Figure 1.

### 3.6. The core diagnostic methods to identify IVS in cats

The neurologists listed NE as the most important diagnostic test for IVS in cats. Furthermore, MRI as well as SB, otoscopy and BP were selected frequently. Veterinary neurologists from NA selected SB more frequently compared with the overall group, otoscopy was selected slightly less often. Otoscopy was mentioned more frequently by EU specialists than by the overall group, and MRI was selected less often. There is large variation in the UK participants compared to the total number of participants. For example, MRI was listed considerably more often in the UK population. The figure can be found in the Supplementary materials.

### 3.7. Therapy of IVS in dogs

A total of 107 participants completed the part of the survey about treatment. Most recommended was the use of intravenous fluid therapy. The most frequently selected intravenous fluid therapy dose was 2 ml/kg/h. In NA, 3 ml/kg/h has been selected with the same frequency as 2 ml/kg/h. Some participants indicated that they adjusted the fluid volumes based on hydration status and did not indicate a fixed intravenous fluid therapy rate.

Maropitant was the most commonly selected antiemetic. The dosage 1 mg/kg once daily was the dose used by most specialists. Rarely, metoclopramide or ondansetron were selected as therapy. Few participants selected all three medications.

Propentofylline was exclusively used by EU specialists. Seventeen chose a dose of 3 mg/kg twice daily. Betahistine was used sporadically. The preferred dosage was 25 mg/kg twice daily.

**Figure 4:**
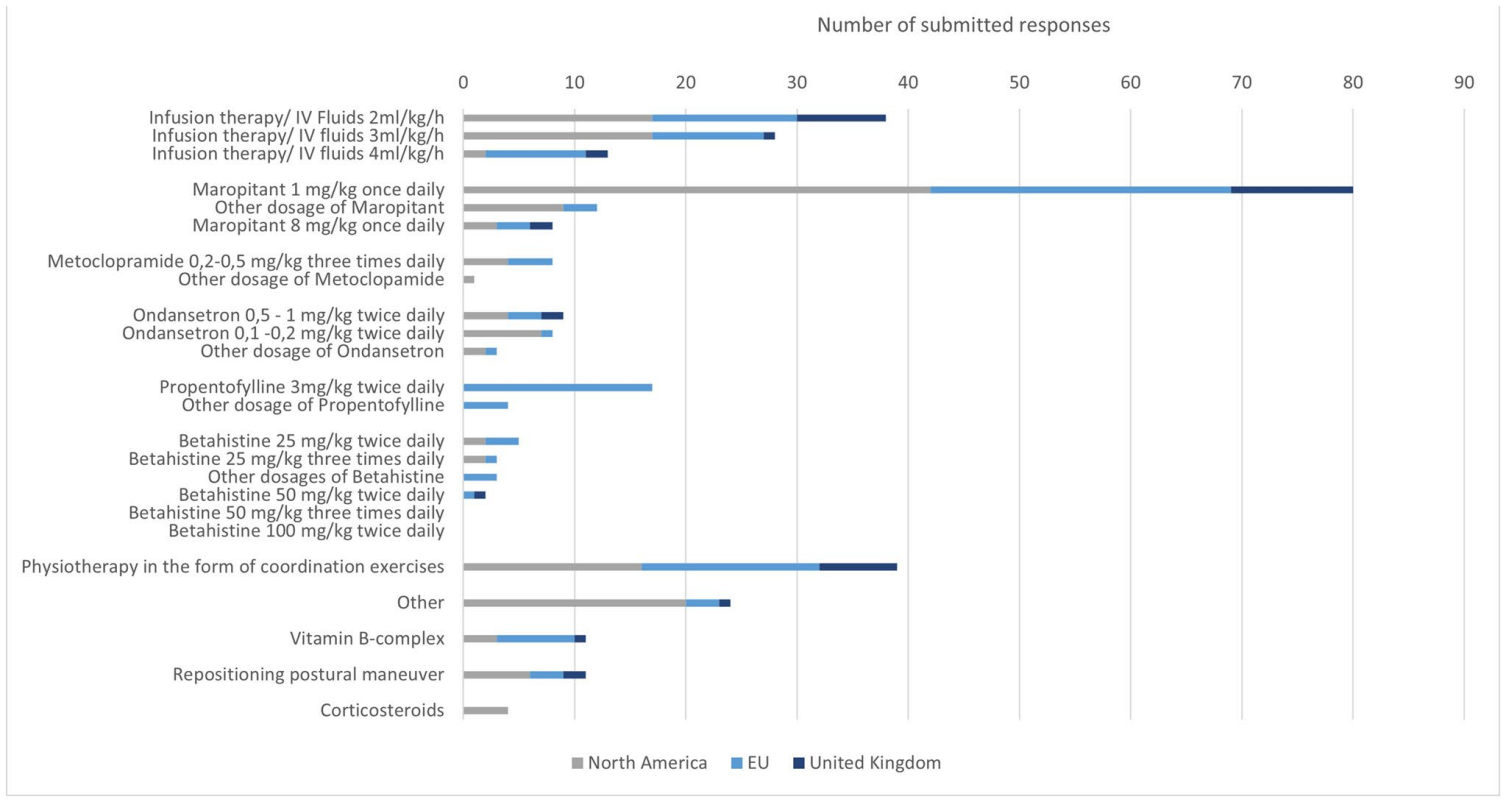
Specialists’ responses regarding treatment preference for IVS in dogs.

Other treatments were rarely mentioned. Physiotherapy seems to be used worldwide to support the improvement of clinical signs. Occasionally, positioning exercises were also mentioned with no geographically prominent prevalence (Figure 4).

### 3.8. Therapy of IVS in cats

Intravenous fluid therapy was selected by 75 participants with a focus on 2 ml/kg/h. The rate 4 ml/kg/h was rarely selected. Some participants mentioned that they will set the rate according to the hydration status of their patients. Antiemetics were frequently recommended in particular maropitant was selected with a favored dosage of 1 mg/kg once daily. Metoclopramide and ondansetron were selected by a few specialists. In very few cases, all three drugs were chosen or two of three were combined. Propentofylline was used in a few cases in the EU with a dosage of 3 mg/kg twice daily. Betahistine was rarely mentioned for the use in cats. Participants from the UK did not choose betahistine, as a possible treatment option for cats. Vitamin B complex was used sporadically by specialists, corticosteroids were occasionally used in NA.

**Figure 5:**
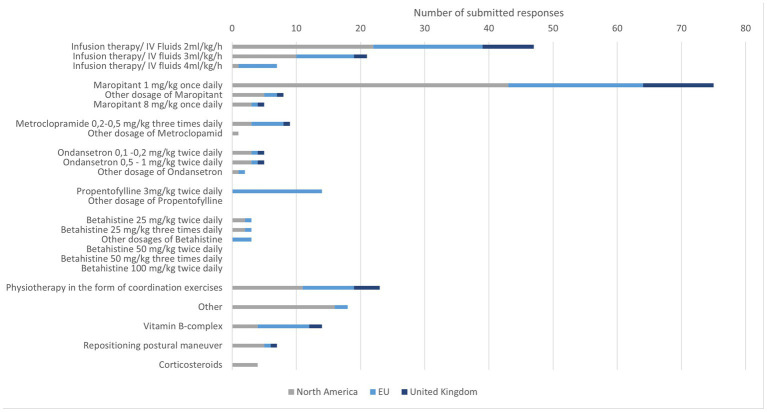
Specialists’ responses regarding treatment preference for IVS in cats.

Physiotherapy with coordination exercises was performed by a third of all participants. Occasionally, positioning exercises were performed in cats. Five participants indicated using both methods (Figure 5).

## 4. Discussion

In recent years, there has been an increasing amount of research on IVS. Multiple authors have suggested IVS to be renamed to benign peripheral vestibular syndrome or facial and vestibular neuropathy of unknown origin (FVNUO) (26–28). However, despite these advancements, there has been a lack of specification in the definition of IVS and limited adaptation of diagnostic and therapeutic approaches. In contrast, human medicine has made more extensive use of diagnostics and has developed specific diagnostic and treatment plans that could potentially serve as a template for veterinary medicine (19, 29–32). The objective of the current study was to provide a comprehensive understanding of how practicing veterinary neurology specialists define IVS, which diagnostic methods they commonly employ, and the therapeutic approaches they utilise. Most veterinary neurologists agreed that IVS should be defined for both cats and dogs, as a condition acute or peracute in onset, improving with a ‘peripheral’ or ‘without central involvement’ vestibular neuroanatomical localisation. Geographical differences were, however, observed in the preferred diagnostic pathways among specialists. For instance, participants from the EU tend to prioritise otoscopic examinations and non-advanced investigations before considering more advanced techniques such as MRI and CSF analysis. Furthermore, variations existed in diagnostic approaches between cats and dogs. For example, in dogs, thyroid levels were often prioritised, while in cats, blood pressure measurements were given more importance compared to canines. Our findings highlight the need for a more standardised approach to the diagnosis and treatment of IVS, considering both geographical and species-specific differences. By identifying these variations, we can better understand the current practices and explore avenues for improving the management of IVS in veterinary medicine.

As aforementioned, IVS was considered by nearly all who answered the question, as a peracute to acute condition of the peripheral vestibular system, which improved quickly. This is aligned with two studies into clinical decision making, which used a statistical modelling approach, considering key clinical features and signalment (33, 34). Both studies showed that in dogs and cats the main difference of IVS compared to other conditions affecting the vestibular system is that they do improve and do not present with Horner’s syndrome (33, 34). In dogs, additional risk factors highlighted in the statistical model were higher age, higher bodyweight, pathological nystagmus, facial nerve paresis and a peripheral neuroanatomical localisation (33). In cats, the additional identified risk factors were being non-purebred and presenting without a history of otitis externa (34). These additional factors are largely in agreement with the results of the survey where IVS was declared a geriatric condition and the neuroanatomical localisation was to the “peripheral” vestibular system.

Possible causes for vestibular dysfunction in dogs and cats can be central or without central involvement (peripheral) in origin. For central causes, differentials include conditions for example inflammatory, such as meningoencephalomyelitis, infections, vascular infarcts, trauma, nutritional or neoplasia. Important differential diagnoses for peripheral causes are otitis media and interna, canine hypothyroidism, infections, neuritis, vascular insults, trauma, and neoplasia (3, 4, 29, 35–38). In addition to the differential diagnoses already listed for dogs, ear polyps should be mentioned additionally for cats (5, 29, 39, 40). To exclude these possible causes of the vestibular clinical signs in cats and dogs, specialists agree on which test should be performed and for the most part agree with the literature. Thus, NE, MRI, SB, CBC, otoscopy, and blood pressure measurement are most frequently mentioned for both species (3, 6, 29, 40). Few specialists use more specific diagnostics such as brainstem auditory evoked responses to detect possible hearing loss, as described for Meniére disease in humans (10–12).

When it comes to the “peripheral” localisation of vestibular disorders, it appears that human medicine offers more specific tests compared to veterinary medicine. These tests enable the determination of the exact affected area, such as identifying which semicircular canal is involved (41, 42). In a publication by Strain et al. in 2010, various diagnostic options for peripheral vertigo in human medicine were listed, along with an explanation of their limitations when applied to veterinary medicine (40). These limitations stem from the practical challenges of handling animals, particularly larger dogs, as well as the significant cost of acquiring specialised equipment. In veterinary medicine, the challenges lie in the fact that animals cannot communicate to the clinician how they experience vertigo or precisely when it is triggered. This lack of direct feedback from the animals makes it challenging to achieve further specification in diagnosing vestibular disorders, in contrast to human medicine. Veterinary medicine must rely on indirect indicators and observations to gather information about the condition and its localisation. The impracticality of certain diagnostic procedures in veterinary settings, combined with the inherent limitations of working with animals as patients, contributes to the difficulties in achieving the same level of specificity in vestibular disorder localisation, as seen in human medicine. Nevertheless, ongoing research and advancements in veterinary neurology continues to address these challenges and improve the diagnostic capabilities for vestibular disorders.

An MRI and CSF analysis were considered by the experts part of the most favoured diagnostic methods. However, EU specialists chose this option less frequently compared to participants from NA or the UK. There could be several reasons for this discrepancy. It is possible that owners in the EU may have limited financial means or lack veterinary insurance coverage to bear the cost of these diagnostic procedures. In addition, some practices or clinics in the EU may not have access to an MRI machine, and owners may have concerns about the potential risks of anesthesia, especially in older animals. Consequently, specialists often rely on neurological examinations, the history and other non-anaesthetic procedures to make a diagnosis, considering the MRI as less essential. Nonetheless, MRI and CSF analysis can be important to rule in or out other potential conditions.

Recent advancements in MRI sequences have challenged the notion that the MRI findings in IVS are always normal. Foth et al. demonstrated a lack of suppression in the inner ear, as evidenced by T2-weighted and FLAIR images, on the side of the lesion in dogs with IVS (27). Another study observed asymmetry of utricular diameters in dogs with IVS compared to a healthy control group (28). Furthermore, Orlandi et al. (26) found increased enhancement of the facial nerve, vestibulocochlear nerve, or both on MRI in some patients previously diagnosed with IVS (26). These recent studies highlight the progress in IVS research, raising questions about the validity of the term “idiopathic” in IVS itself. Orlandi et al. (26) introduced the term “facial and vestibular neuropathy of unknown origin” (FVNUO) as an alternative (26). However, considering that changes in utricular diameters and suppression of the affected inner ear can also occur in IVS patients, which are not covered by the new term, a more suitable umbrella term at this time would be “benign peripheral vestibular syndrome” (27, 28).

In both species, the majority of specialists recommended symptomatic therapy involving intravenous fluid administration and antiemetics. The most frequently suggested infusion rate was 2 mg/kg/h, which corresponds to the lowest maintenance dose for larger dogs without considering potential dehydration and increased fluid loss (43). Some neurologists emphasised the need to adjust the rate of intravenous fluid therapy based on the degree of dehydration in both cats and dogs. The rationale behind the recommendation of intravenous fluid therapy is the proposed association of inadequate inner ear perfusion with IVS, which justifies the use of intravenous fluids to improve perfusion in the affected region of the body. Furthermore, animals experiencing nausea and vomiting exhibit increased water loss and hypersalivation, leading to reduced water intake. Therefore, a pure maintenance dose may not be sufficient, especially when concurrent conditions restrict fluid homeostasis. In such cases, an individualised amount of fluid should be administered to the patient.

In order to address vomiting, antiemetic drugs are commonly utilised. According to the survey, maropitant was the most frequently selected antiemetic in dogs and cats, with a dosage of 1 mg/kg once daily, although it received less mention in cats. Additionally, participants occasionally chose metoclopramide and ondansetron as alternative options. Maropitant has been medically approved for the treatment of motion sickness in cats and dogs, as it acts on the vestibular input to the nucleus of the solitary tract, which can induce nausea and vomiting through the activation of the semicircular canals and the labyrinth (44–46). This mechanism could potentially contribute to the nausea and vomiting experienced by IVS patients. However, unlike vomiting, which operates on an all-or-nothing principle, nausea is a complex sensation with multiple underlying causes (47, 48). A study by Kenward et al. (49) in 2017 demonstrated that maropitant, metoclopramide, and ondansetron were effective in reducing vomiting in dogs, but only ondansetron significantly relieved the signs of nausea (49). Studies on canine patients with vestibular diseases have also shown the efficacy of ondansetron in alleviating nausea (7, 24). In Kenward’s study, maropitant was administered at a dosage of 1 mg/kg, as chosen by most participants in the survey. However, the literature describes a dosage of 8 mg/kg for the treatment of motion sickness, raising the question of whether a higher dosage of maropitant could also effectively alleviate nausea in dogs with IVS (50, 51). These findings suggest the potential consideration of a combined therapy targeting both nausea and vomiting in dogs with IVS, although further investigation is warranted to evaluate this combination and its effects.

In cats, maropitant, metoclopramide, and ondansetron have also demonstrated a reduction in vomiting. However, nausea is only relieved by ondansetron when administered directly with the trigger, as observed in the study involving dexmedetomidine (52–54). When the same dose was administered 30 min prior, there was no difference compared to the control group. Similar results were reported by Lucot et al. in 1989, where a 5-hydroxytryptamine-3-receptor antagonist was administered 20 min before cisplatin, xylazine, or vigorous exercise, resulting in no reduction in vomiting except with cisplatin (55). Therefore, further studies are warranted to test if ondansetron in cats also improves nausea related to vestibular disease.

The divergent effects of these drugs between species can be attributed to the specific receptors involved in central vomiting in the area postrema. In dogs, dopaminergic receptors play a primary role, while in cats, alpha2 adrenergic receptors are predominantly involved (56–58). Consequently, it is important not to extrapolate treatment approaches from dogs to cats. Separate treatment plans need to be developed and implemented for each species.

In the free-text responses, participants reported using dimenhydrinate and meclizine in a small number of cases. Both drugs belong to the class of antihistaminergic and are effective against nausea and vomiting. Dimenhydrinate also has a sedative effect and appears to have similar efficacy to metoclopramide for vertigo-induced nausea in humans (59). The additional sedative effect may help calm the patient initially. Meclizine is also used for vertigo in human medicine and is thought to help with the resulting nausea by inhibiting the visual-vestibular ocular reflex at low stimulation intensity (60). Another drug mentioned by participants in the free-text responses was diazepam. It has a depressant effect on neurons of the vestibular system and thus reduces vertigo (61). However, currently there are no studies yet supporting the usage of the three drugs in companion animals in the context of IVS.

In addition to symptomatic therapy, there are drugs available that specifically target the treatment of vertigo. Betahistine is commonly used in human medicine, particularly for conditions like Menière’s disease and BPPV (21, 22, 62, 63). As an agonist of the histamine H1 receptor and antagonist of the histamine H3 receptor, it reduces activation of the vestibular nuclei and increases cerebral blood flow through vasodilation. This mechanism is believed to improve central compensation for vertigo (21, 64–66). Various dosages ranging from 2 mg/kg to 100 mg/kg have been reported in the literature, each showing positive effects on vertigo but not specifically in patients with IVS (67, 68).

Another potentially helpful drug is propentofylline, which is currently used only in the EU due to a lack of license in NA. In human medicine, it is primarily being researched as a potential therapy for Alzheimer’s disease and other neurodegenerative diseases (69). While there are no studies investigating the use of propentofylline in vestibular disease, animal studies have shown its effects on adenosine levels in the brain, total energy consumption of brain cells, blood circulation, and neuroprotective effects through remyelination of neurons (70–73). These mechanisms of action may potentially aid in vestibular compensation after IVS and thus reduce convalescence time. However, studies on the positive effects of propentofylline as an effective treatment for IVS in cats and dogs are currently lacking.

A small number of participants from NA chose corticosteroids as a treatment. In human medicine, corticosteroids are used in cases of acute vestibular neuritis; however, their positive effect on patient convalescence time remains highly controversial (74, 75). B vitamins have been reported to aid in nerve regeneration following an inflammatory response, resulting in symptomatic improvement. Some veterinary neurologists mentioned the potential benefits of B vitamins in IVS treatment (76). However, the evidence is also lacking in veterinary medicine for this treatment approach.

In addition to drug therapy for vertigo, positioning exercises are also utilised in human medicine, particularly for conditions like BPPV, which commonly affects elderly patients. The vertigo in BPPV is caused by detached mineral crystals from the matrix of the utriculus that enter the semicircular canals. The movement of these otoliths stimulates sensory cells, leading to a mismatch of information between the eyes and the vestibular organ in the brain, resulting in vertigo (13–15, 41, 77). Positioning exercises serve as initial therapy in humans to reposition the otoliths out of the canals, providing immediate relief from clinical signs. Although there are similarities between BPPV and IVS in elderly dogs, it is not proven that IVS has a shared aetiology with BPPV. However, Kraeling in 2014 described the use of positioning exercises in dogs, as a potential extension of IVS therapy, reporting positive outcomes in some patients (2).

Supportive physical therapy, such as coordination and balance exercises, is not commonly practiced but has been employed by some participants in the study. These exercises accelerate brain compensation and can contribute to improvement in vertigo (23, 78). They can be applied regardless of the underlying cause of vertigo, suggesting a potential positive effect in cats and dogs, particularly in cases of prolonged IVS.

The survey provided a comprehensive overview of the current diagnostic and treatment options for IVS in dogs and cats. However, despite the thorough questionnaire, it is possible that certain aspects of diagnosis and treatment were not covered or could benefit from further investigation. Additionally, the variation in the number of participants from different countries may introduce some bias in the utilisation of diagnostics and therapies, especially considering that certain medications may not be available in all countries. Moreover, since the survey targeted veterinary neurology specialists exclusively, there may be other treatment options or variations in diagnostic methods and treatment modalities employed by different veterinary professionals. It is important to note that although more recent imaging and clinical studies exist to better characterise IVS, there is still a significant lack of data regarding its diagnosis and treatment options.

## 5. Conclusion

The study demonstrated that the definition, diagnosis, and treatment of IVS are largely consistent worldwide, with the EU prioritising less frequently advanced imaging. IVS was defined as an acute to peracute peripheral vestibular disorder, and the current survey, along with previous studies, suggests that the clinical course and age should be taken into consideration in the definition. Therefore, IVS can be characterised, as an acute to peracute, improving, non-painful peripheral vestibular disorder that often affects cats of any age and geriatric dogs. Regarding diagnosis, a detailed neurological examination and comprehensive blood tests, including thyroid values, blood pressure, and otoscopic examination, remain crucial. A thorough workup may also involve MRI and CSF analysis to rule out other causes of vertigo. Treatment of IVS typically involves intravenous fluid therapy and the use of an antiemetic, with maropitant once daily being the preferred choice among specialists. However, the authors suggest considering antinausea treatment, since animals may experience nausea even in the absence of vomiting behavior.

This survey-based study provides valuable insights from neurology experts and highlights areas that require further research to bridge the gap between theory and practice. There is solid evidence for ondansetron as antinausea medication, but there remains a lack of research on the efficacy of betahistine, propentofylline, antihistamines (such as dimenhydrinate and meclizine), diazepam, and exercise therapy in reducing the recovery time of IVS.

## Data availability statement

The original contributions presented in the study are included in the article/Supplementary material, further inquiries can be directed to the corresponding authors.

## Author contributions

AM: Conceptualization, Data curation, Formal analysis, Investigation, Methodology, Project administration, Visualization, Writing – original draft, Writing – review and editing. HS: Conceptualization, Supervision, Writing – review and editing. HV: Conceptualization, Project administration, Supervision, Validation, Writing – review and editing.
