# Supplementary material for: Current definition, diagnosis, and treatment of canine and feline idiopathic vestibular syndrome
Source: Front Vet Sci. 2023 Sep 22;10:1263976. doi: 10.3389/fvets.2023.1263976 (PMC10556701; doi:10.3389/fvets.2023.1263976)
Supplement: Supplementary file 1 [file Data_Sheet_1.pdf]

Supplementary Material

Current definition, diagnosis and treatment of canine and feline idiopathic vestibular syndrome.

A. M. Mertens[1,2], H. C. Schenk[2], H. A. Volk[1*]

[1]University of Veterinary Medicine Hannover, Department of Small Animal Medicine and Surgery, Hanover, Germany

[2] Department of Neurology/ Neurosurgery, Tierklinik Lüneburg, Lüneburg, Germany

*** Correspondence:**

Holger Volk

holger.volk@tiho-hannover.de

Klinik für Kleintiere
Stiftung Tierärztliche Hochschule Hannover
Bünteweg 9
30559 Hannover

1 Supplementary Data

A: Questionnaire

Establishment of a standard of care for the idopathic vestibular syndrome in dogs and cats

General information

1. In which country do you practice veterinary medicine?

Please write your answer here:

2. How long have you practiced veterinary medicine for?
Please write your answer here:

3. Do you have any further qualifications?
Choose one of the following answers

Please choose **only one** of the following:

- Diplomate of ECVN
- Diplomate of ACVIM (Neurology)
- Diplomate of ACVIM (Internal Medicine)
- Diplomate of ECVIM
- other qualification
-

4. Have you practiced veterinary medicine in more than one country until now?

If yes, please enter the different countries you have already worked in the comment section.

Please choose **only one** of the following:

- yes
- no

make a comment on your choice here:

-

5. Which description fits your current workplace best?

Please choose **only one** of the following:

- academia
- general practice
- specialist center
- other

6. What is he approximate number of neurological patients per month at your current workplace?

Please write your answer here:

7. What is the approximate number of cases per month with a vestibular disease at your current workplace?

Please write your answer here:

8. What is the approximate number of cases per month with an idiopathic vestibular syndrome at your current workplace?

Please write your answer here:

Diagnosis of idiopathic vestibular syndrome

1. Please describe in your own words what the definition of idiopathic vestibular disease is.

Please write your answer here:

2. In addition to an unremarkable general physical examination, which of the following diagnostic steps should be performed for patients to confirm a diagnosis of idiopathic vestibular syndrome in a **DOG**.Please tick all which applies.

Please choose all that apply:

- neurological examination
- otoscopy
- ocular fundus examination
- hematology
- complete serum chemical profile
- kidney values only
- liver values only

- creatine kinase activity
- c-reactive protein activity
- T4
- T4 and TSH
- blood pressure measurement
- electrocardiogram
- thoracic radiograph
- abdominal radiograph
- bulla radiography
- abdominal ultrasound
- computer tomography (CT)
- magnetic resonance imaging (MRI)
- cerebrospinal fluid examination
- determination of Neospora caninum and Toxoplasma gondii antibodies or PCR
- Other:

3. If you had to choose a minimum of diagnostics, beside a general physical examination, for the work up of a suspected idiopathic vestibular syndrome which ones would you chose in a **<u>DOG</u>**. Please tick a maximum of 5 diagnostic tests.

Please select at most 5 answers.

- neurological examination
- otoscopy
- ocular fundus examination
- hematology
- complete serum chemical profile
- kidney values only
- liver values only
- creatine kinase activity
- c-reactive protein activity
- T4
- T4 and TSH
- blood pressure measurement
- electrocardiogram
- thoracic radiograph
- abdominal radiograph
- bulla radiography
- abdominal ultrasound
- computer tomography (CT)
- magnetic resonance imaging (MRI)
- cerebrospinal fluid examination
- determination of Neospora caninum and Toxoplasma gondii antibodies or PCR
- Other:

4. In addition to an unremarkable general physical examination, which of the following diagnostic steps should be performed for patients to confirm a diagnosis of idiopathic vestibular syndrome in a **CAT**. Please tick all which applies.
Please choose all that apply.

- neurological examination
- otoscopy
- ocular fundus examination
- hematology
- complete serum chemical profile
- kidney values only
- liver values only
- creatine kinase activity
- c-reactive protein activity
- T4
- T4 and TSH
- feline immunodeficiency virus ELISA
- feline leukemia virus ELISA
- blood pressure measurement
- electrocardiogram
- thoracic radiograph
- abdominal radiograph
- bulla radiography
- abdominal ultrasound
- computer tomography (CT)
- magnetic resonance imaging (MRI)
- cerebrospinal fluid examination
- determination of Toxoplasma gondii antibodies or PCR
- Other

5. If you had to choose a minimum of diagnostics, beside a general physical examination, for the work up of a suspected idiopathic vestibular syndrome which ones would you chose in a **CAT**. Please tick a maximum of 5 diagnostic test.
Please select at most 5 answers.

- neurological examination
- otoscopy
- ocular fundus examination
- hematology
- complete serum chemical profile
- kidney values only
- liver values only
- creatine kinase activity
- c-reactive protein activity
- T4

- T4 and TSH
- feline immunodeficiency virus ELISA
- feline leukemia virus ELISA
- blood pressure measurement
- electrocardiogram
- thoracic radiograph
- abdominal radiograph
- bulla radiography
- abdominal ultrasound
- computer tomography (CT)
- magnetic resonance imaging (MRI)
- cerebrospinal fluid examination
- determination of Toxoplasma gondii antibodies or PCR
- Other

Therapy of idiopathic vestibular syndrome

1. Which of the following therapies do you use in your practice for idiopathic vestibular syndrome in a **DOG**? Please tick all which applies.

Please choose all that apply:

- Infusion therapy/ IV fluids, 2 ml/kg/h
- Infusion therapy/ IV fluids, 3 ml/kg/h
- Infusion therapy/ IV fluids, 4 ml/kg/h
- Maropitant 1 mg/kg once daily
- Maropitant 8 mg/kg once daily
- other dosage of Maropitant
- Metroclopramide 0,2 -0,5 mg/kg three times daily
- other dosage of Metroclopramide
- Ondansetron 0,1-0,2 mg/kg twice daily
- Ondansetron 0,5 - 1 mg/kg twice daily
- other dosage of Ondansetron
- Propentofylline 3 mg/kg twice daily
- other doasage of Propentofylline
- Betahistine 25 mg/kg twice daily
- Betahistine 25 mg/kg three times daily
- Betahistine 50 mg/kg twice daily
- Betahistine 50 mg/kg three times daily
- Betahistine 100 mg/kg twice daily
- other dosages of Betahistine
- Vitamin B-complex
- Corticosteroids
- physiotherapy in the sense of coordination exercises
- repositioning postural maneuver
- other:

2. Which of the following therapies do you use in your practice for idiopathic vestibular

syndrome in a **CAT**? Please tick all which applies.
Please choose all that apply:

- Infusion therapy/ IV fluids, 2 ml/kg/h
- Infusion therapy/ IV fluids, 3 ml/kg/h
- Infusion therapy/ IV fluids, 4 ml/kg/h
- Maropitant 1 mg/kg once daily
- Maropitant 8 mg/kg once daily
- other dosage of Maropitant
- Metroclopramide 0,2 -0,5 mg/kg three times daily
- other dosage of Metroclopramide
- Ondansetron 0,1-0,2 mg/kg twice daily
- Ondansetron 0,5 - 1 mg/kg twice daily
- other dosage of Ondansetron
- Propentofylline 3 mg/kg twice daily
- other doasage of Propentofylline
- Betahistine 25 mg/kg twice daily
- Betahistine 25 mg/kg three times daily
- Betahistine 50 mg/kg twice daily
- Betahistine 50 mg/kg three times daily
- Betahistine 100 mg/kg twice daily
- other dosages of Betahistine
- Vitamin B-complex
- Corticosteroids
- physiotherapy in the sense of coordination exercises
- repositioning postural maneuver
- other:

2 Supplementary Table

	started survey	submitted a definition of IVS	submitted diagnostic methods	submitted treatment modality
**all participants**	177	102 (58 %)	112 (63 %)	107 (60 %)
**North America (USA, Canada)**	62	53 (85 %)	59 (90 %)	55 (89 %)
**Europe (Germany, Switzerland, Italy, Portugal, Spain, Poland, France, Netherlands, Belgium, Sweden, Norway)**	41	34 (83 %)	37 (90 %)	36 (88 %)
**United Kingdom**	22	15 (68 %)	16 (73 %)	16 (73 %)

Supplementary Table 1. Demographics of participants by region and survey section completion in absolute numbers and as a percentage of the total number of participants in each region

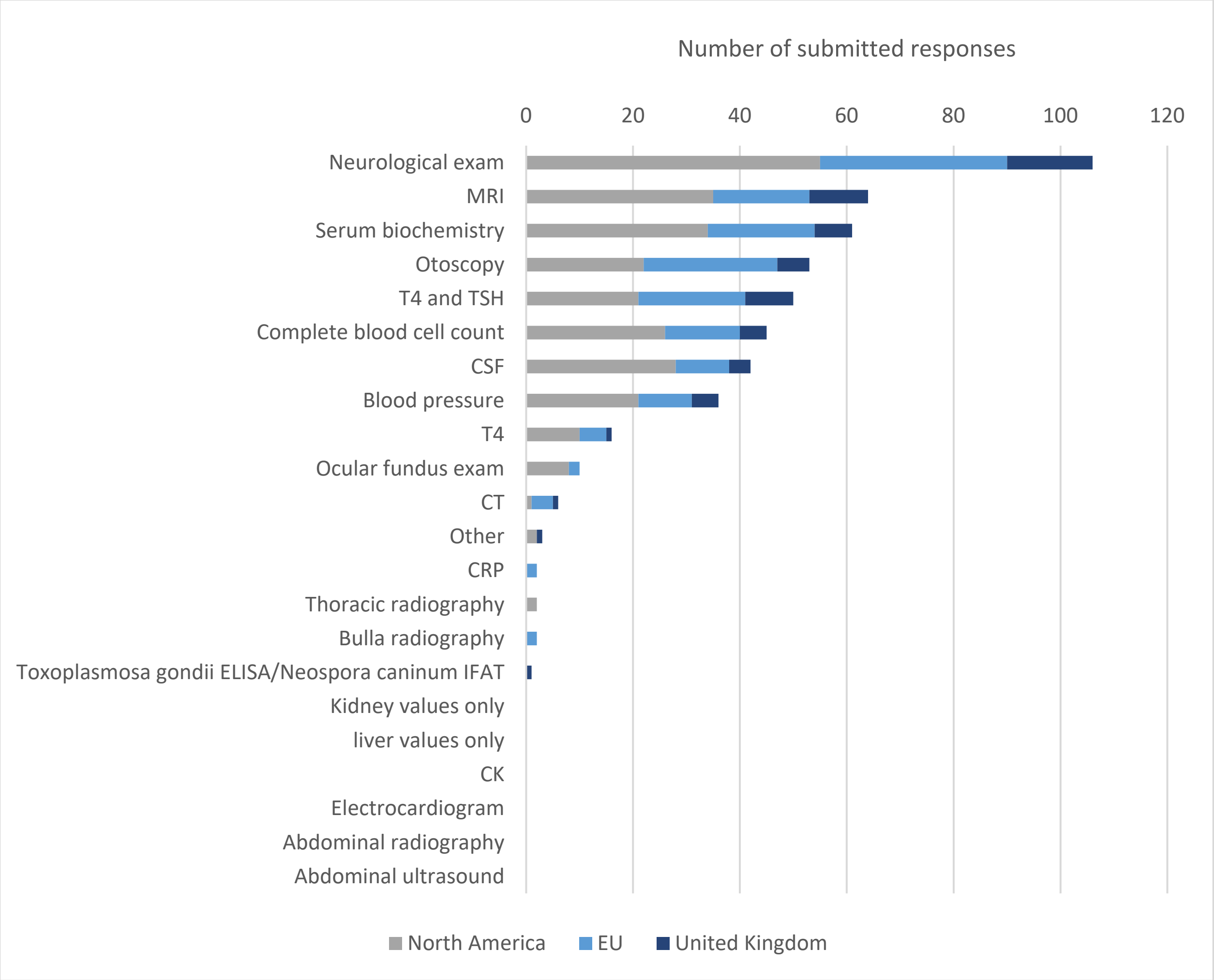

Supplementary Figure 1: Core diagnostic methods to identify IVS in dogs. (MRI = magnetic resonance imaging, CSF cerebrospinal liquid examination, T4 = thyroxine, TSH = thyroid-stimulating hormone, CT = computer tomography, CRP = C-reactive protein, ELISA = enzyme-linked immunosorbent assay, IFAT = Immuno-Fluorescence-Antibody-Test, CK = creatine kinase)

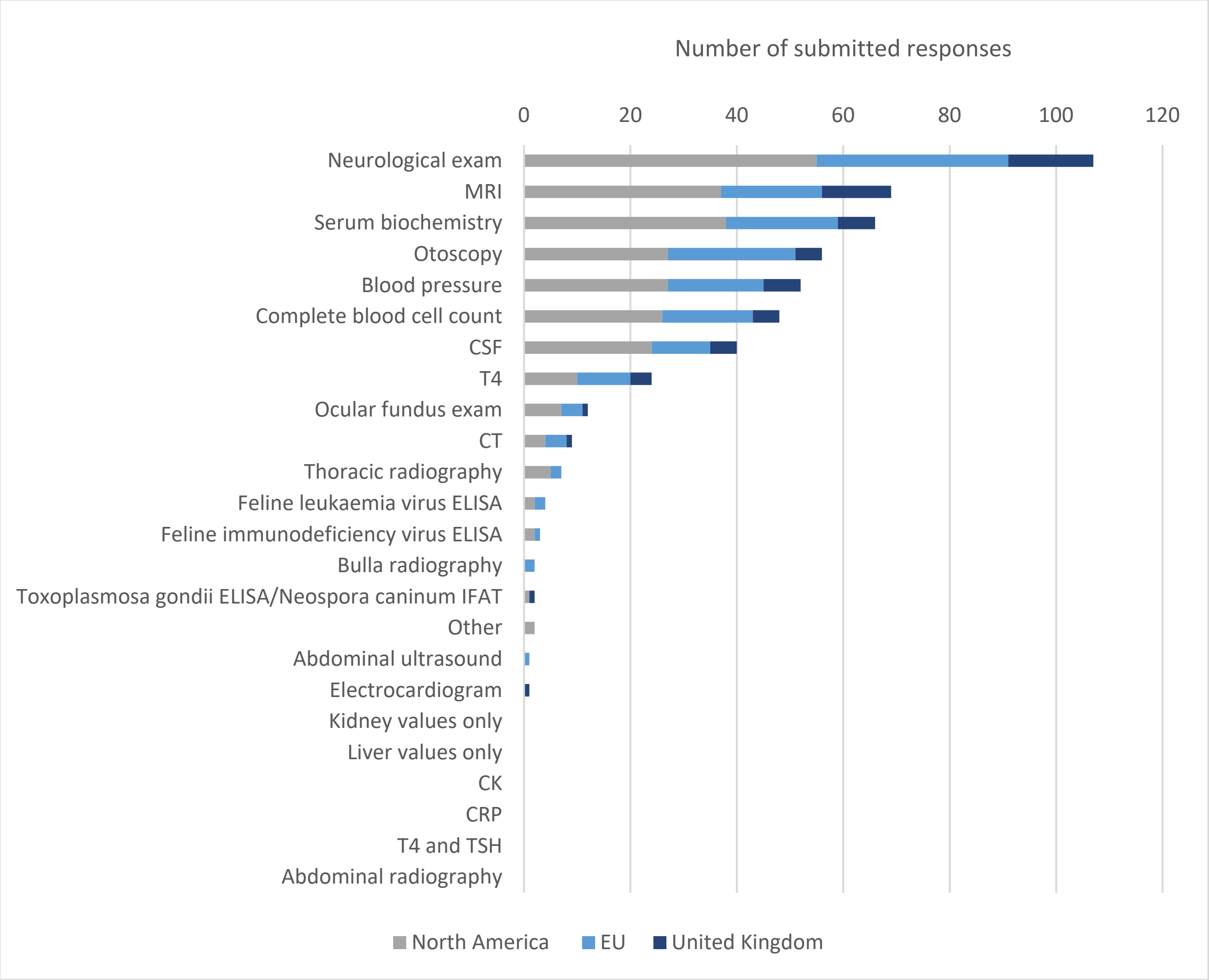

Supplementary Figure 2: Core diagnostic methods to identify IVS in cats. (MRI= magnetic resonance imaging, CSF= cerebrospinal liquid examination, T4= thyroxine, FeLV = feline leukaemia virus, FIV = feline immunodeficiency virus, ELISA= enzyme-linked immunosorbent assay, CT= computer tomography, IFAT = Immuno-Fluorescence-Antibody-Test, TSH= thyroid-stimulating hormone, CRP = -reactive protein, CK= creatine kinase)
